# The Potential Role of a Surface-Modified Additive-Manufactured Healing Abutment on the Expression of Integrins α2, β1, αv, and β6 in the Peri-Implant Mucosa: A Preliminary Human Study

**DOI:** 10.3390/life12070937

**Published:** 2022-06-22

**Authors:** Leandro Amadeu Roth, Marta Ferreira Bastos, Marcelo A. Melo, Valentim A. R. Barão, Raphael C. Costa, Gabriela Giro, João Gabriel Silva Souza, Kinga Grzech-Leśniak, Jamil Awad Shibli

**Affiliations:** 1 Department of Periodontology, Dental Research Division, Guarulhos University, Guarulhos 07023-070, SP, Brazil; rothleandro@gmail.com (L.A.R.); mar_odonto@hotmail.com (M.A.M.); gabi.giro@gmail.com (G.G.); jgabriel.ssouza@yahoo.com.br (J.G.S.S.); or jshibli@ung.br (J.A.S.); 2 Postgraduate Program in Aging Sciences, Universidade São Judas Tadeu, Rua. Taquari, 546, São Paulo 03166-000, SP, Brazil; martafbastos@gmail.com; 3 Department of Prosthodontics and Periodontology, Piracicaba Dental School, University of Campinas—UNICAMP, Piracicaba 13414-903, SP, Brazil; vbarao@unicamp.br (V.A.R.B.); raphaelcavalcante_@hotmail.com (R.C.C.); 4 Dental Science School (Faculdade de Ciências Odontológicas—FCO), Av. Waldomiro Marcondes Oliveira, 20-Ibituruna, Montes Claros 39401-303, MG, Brazil; 5 Department of Oral Surgery, Wroclaw Medical University, 50-367 Wroclaw, Poland; 6 Department of Periodontics, School of Dentistry, Virginia Commonwealth University, Richmond, VA 23298, USA

**Keywords:** integrins, dental implants, peri-implant mucosa, gene expression

## Abstract

The stability of peri-implant soft tissues is essential for long-term success. Integrins play a vital role in biological processes through developing and maintaining cell interactions; however, few studies have evaluated the effects of modifications to abutment surfaces on cell adhesion across integrin expression. Therefore, this pilot study assessed the influence of different surface topographies of titanium healing abutments prepared by additive manufacturing (AM) on the gene expression levels of the integrin subunits α2, β1, αv, and β6 in the human peri-implant mucosa. Thirteen healthy adults were included. Depending on the number of required implants, the subjects were distributed in different groups as a function of healing abutment topography: **group 1** (fully rough surface); **group 2** (upper machined + lower rough); **group 3** (rough upper surface + lower machined); **group 4** (fully machined). A total of 40 samples (*n* = 10/group) of the peri-implant mucosa around the abutments were collected 30 days after implant placement, and subsequently, the gene expression levels were evaluated using real-time PCR. The levels of gene expression of β1-subunit integrin were upregulated for individuals receiving fully rough surface abutments compared with the other surface topographies (*p* < 0.05). However, the healing abutment topography did not affect the gene expression levels of the α2, αv, and β6 integrin subunits in the human peri-implant mucosa (*p* > 0.05). This preliminary study suggested that controlled modifications of the surface topography of titanium healing abutments produced by AM may influence the quality of the peri-implant mucosa in the early stages of the soft tissue healing process.

## 1. Introduction

Dental implants placed in the alveolar bone trigger processes to repair bone tissue and the oral mucosa. In the healing phase, dental implants can be entirely covered by mucous tissue or be connected to the oral cavity with a healing abutment that promotes changes in the surgical wound healing process in either mucosal or bone tissue [1,2]. For example, in cases where the dental implant is placed with a cover screw and wholly covered with primary mucosal closure (i.e., closure, suture, first intention healing), the mucosal tissue will rapidly heal with minimal granulation tissue formation. However, when a healing abutment or a permanent prosthetic abutment and restoration are immediately installed, the healing response of the mucosal tissue will differ from that associated with implant coverage [3]. A blood clot is formed between the healing abutment or the prosthetic abutment and the mucosal tissue [2].

Epithelial tissue cells migrate toward the prosthetic implant/abutment during the healing phase, flatten along the surface, and create a peri-implant epithelium similar to the junctional epithelium (JE). The so-called biological seal protects the underlining bone tissue from being resorbed [4]. The JE attachment to the implant differs from the tooth [5]. Twenty-four hours after the healing process begins, wound margin epithelial cells dissolve the attachment of the hemidesmosomes and exhibit the first signs of migration. Within 48 h, the proliferation occurs, and epithelial cells migrate through the provisional fibrin–fibronectin matrix until they contact cells from the other side of the lesion. This migration is a complex process that depends on matrix-type receptors. Re-epithelialization also depends on proteolytic enzymes, including plasmin and matrix metalloproteinases (MMPs). These enzymes support cell migration at various levels by acting on the breakdown of the provisional matrix, which consequently promotes epithelial cell attachment loss, and also by activating growth factors, such as tumor growth factor (TGF)-β1 and epidermal growth factor (EGF) [6].

Integrins play an essential role in all processes based on developing and maintaining cell–cell and cell–matrix interactions. These include the attachment of basal epithelial cells to the basal membrane, the attachment of epithelial cells to each other, proliferation, differentiation, apoptosis, migration, and wound healing [5,6]. Integrins are expressed in various cell types such as epithelial cells, fibroblasts, osteocytes, endothelial cells, leukocytes, lymphocytes, and platelets. Immunohistochemical studies have shown that five integrins subunits, α2, α3, α6, β1, and β4, are always expressed in all epithelial cells, regardless of whether the tissue is healthy, inflamed, or healing [7]. Integrin αvβ6 is an attachment protein for epithelial cells that is absent in most average healthy epidermis and oral mucosa. Still, it is peculiarly expressed in the JE and the oral gingival epithelium. In vitro studies have shown that αvβ6 binds to the enamel–cementum junction by interacting with fibronectin, tenascin, vitronectin, and latent TGF-β1 [8]. Other studies have suggested that the primary function of αvβ6 in vivo may not be strictly related to cell adhesion but also to its ability to activate latent TGF-β1. Little is known about integrin expression in the peri-implant epithelium (PIE), so it is possible to hypothesize that lowered attachment could contribute to inflammatory lesions and bone loss around implants [5].

Dental implants establish a transmucosal connection between the external environment and the peri-implant tissues. Forming an adequate and stable barrier capable of biologically protecting peri-implant structures is paramount to preventing microbial infiltration. This is a critical part of the soft tissue integration process and can be influenced by the material type (e.g., chemical composition), surface topography, and abutment connection types [1,9]. Regarding surface topography, there is no consensus on whether abutments with smooth or rough surfaces provide the optimal relationship with the contouring soft tissue. For instance, modified implant surfaces improve fibronectin reactivity and cell proliferation and establish a more significant binding of collagen fibers during the early stages of healing [9,10,11,12]

On the other hand, clinically, the dimensions of the biological width were similar across turned, oxidized, and acid-etched abutments. Still, longer JE was noted for angled and rough surfaces [13]. Modifications in healing abutments and implant surfaces are intended to provide an ideal topography with the potential to modulate the cellular behavior of the peri-implant epithelium, being a mechanical barrier to the connective tissue and improving attachment at the molecular level through the action of integrins.

Additive manufacturing (AM) is an emerging process for industry components, machines, cars, airplanes, biomaterials, and medical devices. AM produced titanium (Ti) and Ti-based alloy implant materials using a specific technique called direct metal laser sintering (DMLS). This technique provides an exciting venue for treating implant and abutment surfaces. DMLS is based on a high-energy focused laser beam aimed at a specific region of a thin layer of metal powder that directly melts following a sliced 3D computer-aided design model. The surface topography showed properties comparable with the wrought samples and, therefore, could positively influence the biological behavior of the human soft and hard tissues [11,14,15].

However, few molecular studies [11,15,16] have evaluated the effects of different surface topographies of abutment healing on soft tissue implant prognosis. Therefore, we sought to assess whether or not different healing abutment surface topographies would affect the gene expression levels of the integrin subunits α2, β1, αv, and β6 in human peri-implant mucosa at short time healing (30 days). The hypothesis is that the implant surface topography will positively impact the integrins’ gene expression levels.

## 2. Materials and Methods

### 2.1. Study Population, Inclusion, and Exclusion Criteria

Subjects seeking dental implant placement in the posterior region who were referred to the Oral Implantology Clinic of Guarulhos University (Guarulhos, SP, Brazil) were selected. The inclusion criteria used in this study were: aged ≥ 30, sufficient residual bone ridge for installing a 3.75 mm diameter dental implant and at least 10 mm length, and a wide range of keratinized tissue (>4 mm). The exclusion criteria were: insufficient bone volume, type 4 bone quality, a high degree of bruxism, smoking, excessive alcohol consumption, radiotherapy, chemotherapy, liver disease, blood dyscrasias, nephropathy, immunosuppression, chronic use of corticosteroids, pregnancy, lactation, autoimmune diseases, poor oral hygiene including oral cavity lesions, or the presence of generalized periodontitis on phases 3 or 4 (i.e., clinical attachment level higher than 5 mm and bleeding on probing in more than 30% of subgingival sites). The Ethics Committee approved the experimental protocol of the University of Guarulhos (#113/2008), and the patients signed an informed consent form.

### 2.2. Experimental Design and Additive Manufactured (DMLS) Healing Abutment

**Figure 1:**
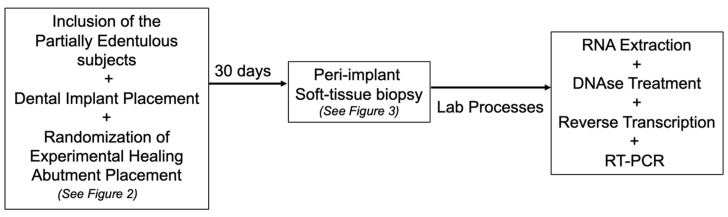
The study outline.

The study protocol was described earlier [11] (Figure 1). The AM process [14] produced experimental healing abutments (4 mm diameter and 3 mm height). Briefly, AM titanium healing abutments were made of Ti-6Al-4V powders with particle sizes of 25–45 μm and sintered together, layer by layer, through an AM process. AM used an ytterbium equipment system with a spot size of 0.1 mm, a wavelength of 1054 nm, and continuous power of 200 W at a scanning rate of 7 m/s laser (Eosynt^®^270, EOS, Munich, Germany). After the AM process, to remove non-fused titanium particles, the healing abutments were sonicated for 5 min in distilled water at 25 °C, immersed in NaOH (20 g/L) and hydrogen peroxide (20 g/L) at 80 °C for 30 min, and then further sonicated for 5 min in distilled water. Following this process, an organic acid treatment was performed in a mixture of 50% oxalic acid and 50% maleic acid at 80 °C for 45 min, followed by washing for 5 min in distilled water in a sonic bath. Grade 4 Ti healing abutments produced using a milling machine were considered the control group.

**Figure 2:**
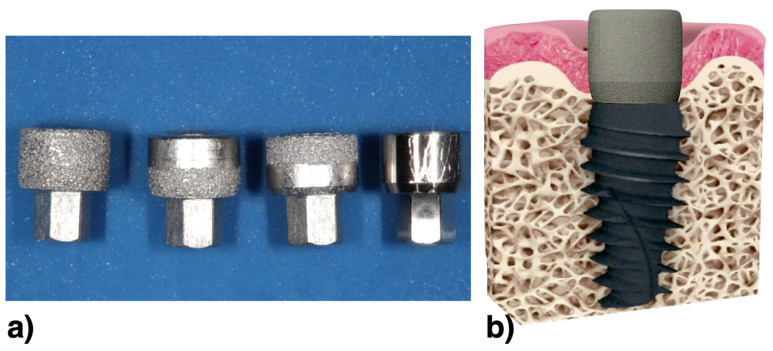
(**a**) The clinical view of the four types of experimental healing abutments: AM abutment with a fully rough surface; AM abutment at a bottom area with the upper part as machined; AM abutment at the upper area and the as-machined surface at the base area; fully as-machined—smooth surface (control group); (**b**) Illustrative drawing showing the healing abutment placed on the dental implant. The region of interest was the peri-implant soft tissue.

Therefore, the groups of the study were composed of 4 different abutment surfaces (*n* = 10 samples per group) installed randomly (using a computer-generated table) into placed implants using software to create a table, and they were described above (Figure 2):

Group 1: AM abutment with a fully rough surface;

Group 2: AM abutment at a bottom area with the upper part as machined;

Group 3: AM abutment at the upper area and the as-machined surface at the base area;

Group 4: Fully as-machined—smooth surface (control group).

In this study protocol, 13 patients received at least 1 internal hexagon implant of 3.75 mm diameter and different lengths (between 10 and 13 mm) installed in the posterior regions of the maxilla and mandible. The implants were installed according to the technique recommended by the manufacturer. After implant placement and torque checking (minimum 30 N/cm), the implants immediately received their respective healing abutments, providing clinical conditions for healing at single-stage implant surgery.

**Figure 3:**
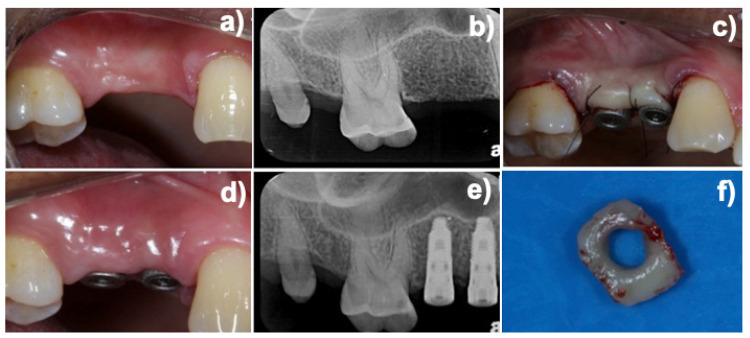
(**a**) View of the edentulous area before dental implant placement and (**b**) the radiographic preoperatory view. (**c**) Experimental healing connected to the implant after suture and (**d**) after 30 days of healing. (**e**) Radiographic view of the connected healing abutments and (**f**) soft tissue biopsy.

After installing the experimental healing abutments and checking the implant platform adaptation through periapical radiographs, the flaps were sutured around the healing abutments with 5.0 nylon (Figure 3). All patients received supragingival biofilm removal and were included in a weekly oral hygiene assessment and instruction maintenance program. After a 30-day healing period, a peri-implant mucosa biopsy was performed with a 15c scalpel around the healing abutments as previously described [11,12]. The mean dimensions of the peri-implant biopsies were 2.0 mm thick and 3.0 mm high.

### 2.3. Abutment Surface Topography Characterization

AM and machined Ti discs (Ø = 8 mm × 2 mm) were used for surface characterization (*n* = 3). Two- and three-dimensional images were acquired at 50× magnification and assessed using confocal laser scanning microscopy (CLSM; VK-X200 series, Keyence, Japan) (Costa et al., 2020). CLSM micrographs were obtained from each sample and processed with the VK Analyzer v3.3.0.0 software (Keyence, Osaka, Japan) to obtain the surface roughness parameters (average roughness—Ra; root mean square roughness—Rq, and average maximum height of the profile—Rz) [17].

### 2.4. Gene Expression Evaluation

#### 2.4.1. RNA Extraction

Immediately after the biopsies were performed, the peri-implant mucosal tissue samples were packed in an RNAlater^®^ solution (Ambion Inc., Austin, TX, USA) to prevent RNA degradation. Samples were incubated at 4 °C for 24 h and then stored at −20 °C until extraction. First, the later RNA solution was aspirated, and the tissue was packaged in liquid nitrogen for shredding. The triturated sample was then placed in TRIZOL reagent (Gibco BRL, Life Technologies, Rockville, MD, USA), homogenized for 30 s, and incubated for 5 min at room temperature. After this period, chloroform (Sigma, St. Louis, MO, USA) was added, and the samples were vortexed and centrifuged at 11,500 rpm for 15 min at 4 °C. The aqueous portion was transferred to another tube to which isopropanol was added, and then it was stirred, incubated for 20 min at −20 °C, and centrifuged as described above. The RNA samples were resuspended in approximately 50 µL of diethylpyrocarbonate (DEPC) treated water and stored at −70 °C. Finally, the RNA concentration was determined using a spectrophotometer. Then, 1 µg of total RNA was evaluated for quality by 1% agarose gel electrophoresis.

#### 2.4.2. DNAse Treatment

Total RNA samples were treated to dispose of DNA residue with DNAse (DNA-free TM, Ambion Inc., Austin, TX, USA) as recommended by the manufacturer. Based on the previously evaluated RNA concentration, the buffer solution and DNAse turbo were added to the tubes with the extracted RNA. After shaking and centrifugation, samples remained incubated at 37 °C for 30 min. Finally, the inactivator was added, and the solution was stirred and centrifuged. Total RNA was again quantified using a spectrophotometer.

#### 2.4.3. Reverse Transcription

A total of 2 µg of the total DNA-free RNA sample was used for the cDNA synthesis. The reactions were performed to a final volume of 30 µL using the First-Strand cDNA Synthesis Kit (Roche Diagnostic Co., Indianapolis, IN, USA), following the manufacturer’s recommendations. Initially, the samples were incubated for 10 min at 25 °C and then for 60 min at 42 °C. After the second incubation step, the samples were incubated for 5 min at 95 °C and then for 5 min at 4 °C for cooling. The reagents used and their respective concentrations were buffer solution (1×), MgCl_2_ (5 mM), deoxynucleotides (1 mM), randomized primers (3.2 µg), RNAse inhibitor (50 U), and AMV reverse transcriptase (20 U).

### 2.5. Real-Time PCR (RT-PCR) Gene Expression Analysis

#### 2.5.1. Primer Design

The GAPDH (glycerin-aldehyde-3-phosphate-dehydrogenase, reference gene) primers, α2, β1, αv, and β6, were designed with the aid of software developed explicitly for LightCycler priming (Roche Diagnostics GmbH, Mannheim, Germany). All primers were checked for specificity with melting curve analysis, always positive and negative controls. Table 1 shows the primer sequence, reaction profile, and amplicon size.

#### 2.5.2. Reaction Optimization

The efficiency of each gene was optimized before the start of the reactions. Concentrations ranging from 2.5 to 5 uM for each pair of primers were used to determine under which conditions the response presented the best efficiency; as suggested by the equipment manufacturer, 5 µM was chosen.

#### 2.5.3. RT-PCR Reactions

RT-PCR reactions were performed with the LightCycler system (Roche Diagnostics GmbH, Mannheim, Germany) using the FastStart DNA Master SYBR Green I kit (Roche Diagnostics GmbH, Mannheim, Germany). The reaction profile was determined following the protocol suggested by the equipment manufacturer. Water was used as a negative control for each analysis, and the reaction product was quantified using the manufacturer’s software (LightCycler Relative Quantification Software 96—Roche Diagnostics GmbH, Mannheim, Germany). GAPDH gene expression levels were used as the reference (housekeeping) to normalize values.

### 2.6. Statistical Analysis

Initially, the data were analyzed for normality with the Shapiro-Wilk test, and nonparametric statistical methods were used when non-normality of values was detected. Fisher’s exact test assessed differences in gender frequency. One-way ANOVA evaluated the ages of the individuals included in the study. Demographic data were presented as mean and standard deviation, and gender was expressed in percentage. Comparisons in gene expression of integrins α2, β1, αv, and β6 were performed using the Kruskal–Wallis test, and comparisons of significant differences between group pairs were performed using Dunn’s test. Results were expressed as median, minimum, and maximum values. Statistical analysis was performed using Prism 6.0 software (GraphPad Software Inc., San Diego, CA, USA). For all analyses, the significance level was set at 5%.

## 3. Results

The study population consisted of 13 individuals (5 males and 8 females), with a mean age of 39 years. All individuals included in the study had clinical characteristics of periodontal health (data not shown).

**Figure 4:**
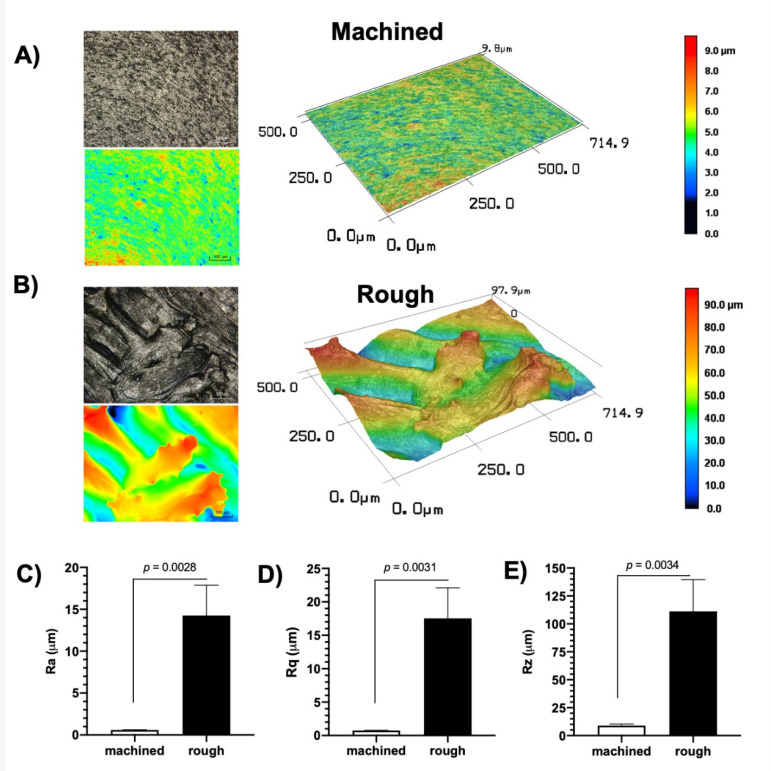
Abutment surface topography characterizations. Representative (**A**) 2D and (**B**) 3D images from each group were obtained by confocal laser scanning microscopy (CLSM; 150× magnification). The surface roughness parameters, i.e., Ra (**C**), Rq (**D**), and Rz (**E**), were calculated from CLSM two-dimensional images. Data are expressed as mean ± standard deviation (*n* = 3). *p*-value was obtained using the *t*-test.

Regarding abutment surface characterization, the machined surfaces (control group) presented longitudinal grooves and a more homogeneous surface. Meanwhile, the AM process’s rough surface (test group) exhibited irregular blasted facets with nonuniform aggregates and greater vertical discrepancies. These surface characteristics could be visualized in the 2D and 3D images obtained by CLSM (Figure 4A,B). The larger areas can be noted in black and dark blue or red and orange, representing deeper valleys and higher peaks. Moreover, all surface roughness parameters—Ra (Figure 4C), Rq (Figure 4D), and Rt (Figure 4E)—of the rough surfaces were higher than those observed for the machined surface (*p* < 0.05), confirming the irregular profile demonstrated by the CLSM micrographs. In fact, the rough surfaces demonstrated Ra = 14.23 μm (±3.61), Rq = 17.53 μm (± 4.55), and Rz = 111.19 μm (±28.34), with values ~25× higher than the control (Ra = 0.58 μm, ±0.03; Rq = 0.74 μm, ±0.04; Rz = 8.97 μm, ±1.34).

**Figure 5:**
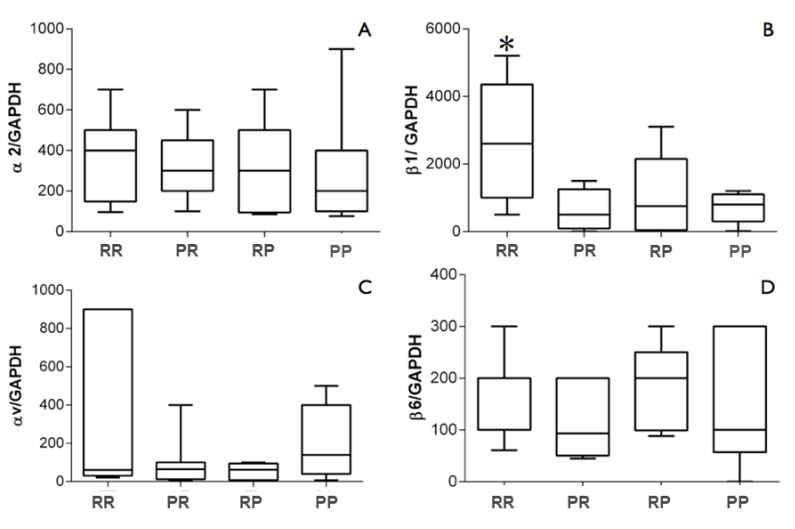
Gene expression levels of integrins (**A**) α2, (**B**) β1, (**C**) αv, and (**D**) β6 in comparison with the expression of the reference gene GAPDH (glycerin-aldehyde-3-phosphate-dehydrogenase) in the peri-implant tissues. * Statistical significance was detected by Kruskal–Wallis test, and comparisons of significant differences between group pairs were performed using Dunn’s test (*p* < 0.05).

Two samples (from groups 1 and 4) that did not present adequate quality to proceed with the analyses were excluded during the RNA extraction. In the RT-PCR step, two other samples (from groups 2 and 3) did not show reference gene expression (GAPDH) and were excluded from the other analyses. Therefore, 9 samples were analyzed per group (Table 2). For the analysis of gene expression of integrin subunits, the different surface topographies of the titanium healing abutments did not affect the levels of α2, αv, and β6 (*p* > 0.05). However, the fully rough abutment (group 1) upregulated almost 3× the gene expression of the subunit β1 compared with groups 2 (polished top + rough bottom) (*p* = 0.006), 3 (rough top + polished bottom) (*p* = 0.031), and 4 (fully polished) (*p* = 0.01) (Figure 5).

## 4. Discussion

The present study determined whether the surface topography of healing abutments manufactured by AM would influence the gene expression of the integrin subunits α2, β1, αv, and β6 in human peri-implant mucosa after a short healing period. Upregulation of 3× the integrin subunit β1 was observed in the tissues collected from individuals receiving a fully rough abutment surface compared with the other surface topographies. However, the healing abutment surfaces did not affect the gene expression of the subunits α2, αv, and β6. This observation confirms a previous preclinical study [18] demonstrating that hydrophilic surfaces influenced the integration of soft and hard tissues through histomorphometric and immunohistochemical analyses. Later, the same group [10] studied humans and observed that hydrophilic surfaces improved the adhesion of peri-implant tissues, conferred through histological examinations in epithelial contact subepithelial connective tissue contact to the abutment surface, showing better histological features when compared with the standard as-machined Ti surface.

Integrins are a family of cellular receptors critical in cell attachment, the regulation of keratinocyte function, healing, and early intracellular signaling in response to extracellular matrix components [19,20]. The interaction between integrins and ECM components plays a central role in all processes that form the basis for wound re-epithelialization and JE regeneration [7]. Atsuta et al. [21] proposed the denomination peri-implant epithelium (PIE) to distinguish the JE around dental implants from those around the tooth. The authors demonstrated through an animal model that the epithelial cell attachment to the implant surface is located in the apical portion of the PIE. The JE is derived from the reduced enamel epithelium, whereas the PIE results from the oral mucosa epithelium [22,23]. Surprisingly, to date, no studies have evaluated epithelial attachment through the gene expression of integrins in peri-implant tissues. This precluded a direct comparison of the data obtained in the present study with those available in the literature.

On the other hand, most human gingival tissue studies analyzed the presence of integrins in gingival tissue using the immunohistochemical or immunofluorescence methodology [11,19,24,25,26]. Hormia et al. [24] reported that the integrin subunit β1 was expressed in the basal cell membrane of the gingival epithelium, along with the JE, and in connective tissue cells, including endothelial cells, with most events of cellular attachment to the matrix extracellular factors being mediated by integrin β1 [27]. In addition to the attachment function, the integrin subunit β1 also plays a vital role in maintaining cell–cell interaction [28]. Thus, the increased expression of the integrin subunit β1 observed for the group of individuals who received fully rough surface healing abutments suggests that this type of topography may favor epithelial migration and attachment, promoting improved healing and/or increasing the JE attachment to the titanium surface, at least in the earlier stages of wound healing. The influence of surface roughness on soft tissue attachment has shown contradictory data. While some data showed no effect of surface roughness on such parameters [29], others have demonstrated that optimal soft tissue integration can be achieved with moderately rough surfaces [10,30], which may correlate with our study. We could expect that treated Ti surfaces would affect cell attachment, the healing process, and, consequently, the host response [11,16].

In the present study, no differences were observed in the expression levels of the subunits αv and β6 for the individuals receiving healing abutments with different topographies. However, there are no studies evaluating the presence of integrins in peri-implant tissue, which precludes comparison with the results obtained. Interestingly, Larjava et al. [5] suggested that the primary function of αvβ6 in vivo may not be directly related to epithelial attachment but to its ability to activate latent TGFβ-1, which can also be related to periodontal disease. This cytokine is part of a polypeptide family with several regulatory functions in tissue repair and the immune system [8,20,31]. Koivisto et al. [8] demonstrated that the addition of TGF-β1 to keratinocyte culture promoted a specific increase in αvβ6 expression while levels of other integrins such as α5β1 and αvβ1 remained unchanged. Häkkinen et al. [20] demonstrated the involvement of integrin αvβ6 in the wound-healing process in mouse epidermis. In the same study, the authors used transgenic mouse strains and showed that increased αvβ6 expression was strongly associated with an abnormal healing process and the onset of chronic lesions. Since the inflammatory process is part of the initial stage of the healing process, it is possible that changes in the levels of αvβ6 due to its ability to interact with TGFβ-1 could interfere with the subsequent stages of the healing process. In a study in human and animal models, Ghannad et al. [31] concluded that integrin αvβ6 is expressed in the epithelium sealing around the tooth and plays a central protective role in periodontal disease through TGFβ-1 activation.

Few studies have investigated the effects of different implant surface topographies on the inflammatory process or the nature of cells infiltrating peri-implant tissues. According to An et al. [32], hydrophilic rough surfaces improve epithelial cell proliferation compared with polished surfaces. In contrast, lower protein expressions of rat oral epithelial cells grown, lower cell attachment, migration, and proliferation were noted on the rough surface compared with smooth surfaces [33]

Taking the surface roughness of dental implants and their components into the spotlight, it is worth identifying an optimal implant surface that decreases bacterial attachment and improves mucosal tissue attachment, which remains in debate [12,34,35]. Although previous systematic reviews [3,36] have shown no influence of surface topography on peri-implant bleeding on probing—which might argue no effect on bacterial attachment—and on peri-implant soft tissue healing, inflammation, and maintenance, such data should be taken with caution. Overall, it is well-known that there is a synergic effect of the chemical and physical properties of the Ti implant surface in controlling microbial accumulation [35]. In this line, classic studies have demonstrated increased biofilm formation on transmucosal implant surfaces with higher surface roughness and surface free energy [37,38]. As the long-term survival of dental implants partially depends on the control of bacterial infection in the peri-implant region [12,39,40], long-term clinical studies are necessary for drawing more profound conclusions on whether or not rough transmucosal surfaces might have a detrimental effect on the peri-implant microbial parameters and soft and hard supporting tissues stability.

This preliminary study has some limitations as the expression of integrins was the only marker investigated in an early stage. However, as healing abutments are used temporarily in clinical situations, the experimental model of the present study simulated the behavior of the peri-implant mucosa in prosthetic abutments to evaluate the expression of integrins with different surface topographies 30 days after the abutment installation, as performed in previous studies [10,12,16]. However, the fully rough surface topography that would affect the peri-implant parameters in the long term should be further investigated, including in peri-implantitis situations. Finally, the study’s strength was its evaluation of peri-implant human soft tissue, the identification of the translational effect of the roughness of implant surface in the oral cavity, and the evaluation of the manufacture additive process as a reliable process for producing dental implant abutments.

## 5. Conclusions

In conclusion, within the limitations of this preliminary study, there was an increase in the expression of integrin β1 in the peri-implant mucosa around fully treated rough healing abutments produced by AM in comparison with other surface topographies. Further fundamental clinical studies are needed to better comprehend the peri-implant soft tissue around prosthetic abutments with different surface topographies on periodontal and microbiological parameters.

## Figures and Tables

**Table 1:** The percentages of guanine (G) and cytosine (C) residues and melting temperature (MT) of the GADPH (glycerin-aldehyde-3-phosphate-dehydrogenase) primer.

Gene	Sequence (5′–3′)	Amplification Profile [Temperature (°C)/Time (s)]	Amplicon Size (bp)
	F: AGCCTATTCGAGCTGCC	95/10; 56/5; 72/10	290
α2	R: CAGTGTTGTATGCACTTTCCC		
β1	F: GTAACAATGGAGAGTGCGTC	95/10; 54/10; 72/10	300
	R: GCTCTGCACTGAACACATTC		
αv	F: CACCAACTCCACATTGGTTAC	95/10; 56/7; 72/10	289
	R: CTGCAGTTAAGTTTCTGAGTTTCC		
β6	F: GTACTGCAACTGCACCAC	95/10; 56/7; 72/10	295
	R: GCAGCTCCGTTTAGAGTTAC		
	F: CTGAGTACGTCGTGGAGTC	95/10; 56/5; 72/7	250
GAPDH			
	R: TGATGATCTTGAGGCTGTTGTC		

**Table 2:** The distribution of the experimental healing abutments according to each subject. **Groups 1** (totally rough); **group 2** (lower AM/ upper machined); **group 3** (lower machined/upper AM), and **group 4** (machined). A total of 40 experimental healing abutments were placed. However, after laboratory processing, 9 healing abutments per group were evaluated (* samples not included in the study).

Patients	Age (Years)	Healing Abutments per Patient	Groups			
			1	2	3	4
1	41	3	-	1	1	1
2	33	3	1 *	1	1	-
3	33	6	1	1 + 1 *	2	1
4	41	3	1	1	-	1
5	32	3	1	-	1 *	1
6	31	1	1	-	-	-
7	35	4	1	2	1	-
8	46	3	-	1	1	1
9	45	4	1	1	1	1
10	45	1	-	-	-	1
11	50	4	1	1	1	1 *
12	26	4	1	-	1	2
13	50	1	1	-	-	-
Total	39.07	40	10	10	10	10
